# Postpartum Depression Following a Substance-Exposed Pregnancy: The Role of Age, Race/Ethnicity, and Mental Health History

**DOI:** 10.7759/cureus.115659

**Published:** 2026-09-02

**Authors:** Natalie Aguilar, Miranda C Manzo, Patrick Fakhoury, Jacob Surma, Beth Bailey

**Affiliations:** 1 Pediatrics, Central Michigan University College of Medicine, Saginaw, USA; 2 Obstetrics and Gynecology, Central Michigan University College of Medicine, Saginaw, USA; 3 Anesthesiology, Central Michigan University College of Medicine, Mount Pleasant, USA; 4 Otolaryngology, Central Michigan University College of Medicine, Mount Pleasant, USA; 5 College of Medicine, Central Michigan University College of Medicine, Mt. Pleasant, USA

**Keywords:** maternal mental health, postpartum depression, prenatal substance use, race/ethnicity, substance-exposed pregnancy

## Abstract

Background and objective

Postpartum depression (PPD) affects 13-19% of postpartum individuals and is one of the most prevalent complications of pregnancy, with significant consequences for maternal and child well-being. Research suggests that maternal age, ethnic minority status, and a history of depression are predictors of PPD. Nearly one-third of substance users also experience PPD, yet it is unclear how age, race/ethnicity, and mental health history predict PPD in this already at-risk population. This study aimed to examine the impact of age, race/ethnicity, and depression history on the development of PPD among individuals who used substances during pregnancy.

Methods

A medical chart review was conducted at two university-affiliated pediatric practices in the Midwestern United States. Eligible participants were those with available maternal prenatal and delivery records, pediatric medical records through age three years, and biochemically confirmed prenatal substance use. The primary outcome was PPD, assessed primarily using the Edinburgh Postnatal Depression Scale with a score ≥ 10 through six months postpartum and/or a documented maternal report of a postpartum depression diagnosis or treatment.

Results

Of 255 participants, 30.19% experienced PPD. Private medical insurance holders (a proxy for higher socioeconomic status (SES)) were twice as likely to develop PPD (odds ratio (OR) = 1.96, 95% confidence interval (CI): 1.09-4.28), while marijuana use increased the risk by nearly 3.4-fold (OR = 3.4, 95% CI: 1.66-6.96). Black women were nearly two and a half times more likely than White, non-Hispanic women to develop PPD, and women who reported another race or mixed race were more than four times more likely to develop PPD. A history of mental health conditions predicted a non-significant, fourfold increase in PPD risk. Age was not a significant predictor. Marijuana use and higher SES were the strongest predictors of PPD in this substance-exposed population.

Conclusions

Marijuana use during pregnancy, race/ethnicity, and private insurance status were independent predictors of PPD in this substance-exposed cohort. Age and pre-pregnancy mental health history were not significant in the adjusted analysis. These findings highlight the need for targeted maternal mental health screening and further research on ascertainment differences by SES.

## Introduction

Postpartum depression (PPD) is one of the most prevalent morbidities related to pregnancy and childbirth, affecting an estimated 13% to 19% of women after giving birth [1]. With about four million births per year in the United States, this represents a considerable number of women affected by PPD (approximately 700,000 women annually) [1]. Despite its high prevalence, about 80% of women with PPD may remain undiagnosed and untreated [2]. This is clinically significant, as the risks associated with PPD may have negative outcomes for both the woman and child.

Substance use during pregnancy is a prevalent public health issue that has been widely studied, as there are adverse effects for both the mother and fetus, and it has been associated with an elevated risk of PPD across multiple studies. A survey conducted by the Substance Abuse and Mental Health Services Administration (SAMHSA) showed that about 11.6% of pregnant women smoke tobacco, 5.4% use illicit drugs, and 4.7% smoke marijuana [3]. Substance use and misuse have consistently been associated with an increased risk of developing PPD [4]. A recent meta-analysis showed that 29% of pregnant women who used substances during their pregnancy experienced PPD [4]. Thus, pregnant women who use substances are at an increased risk of developing PPD.

Marijuana is of special note due to its increased use and prominence in the post-legalization era of the last decade, as access expanded through recreational and medical legalization in many U.S. jurisdictions. In parallel, marijuana use during pregnancy has risen, along with perceptions that use during pregnancy is safe. However, emerging evidence shows that cannabis exposure may be associated with postpartum depressive symptoms and PPD, with an increased likelihood of screening positive for PPD among individuals using marijuana during pregnancy [5]. In contrast, other studies evaluating symptoms of depression have not found any clear associations between prenatal marijuana use and changes in depressive symptoms over time, highlighting the complex interplay that exists between mental health status, self-medication with marijuana, and use of multiple substances simultaneously [6]. These conflicting results underscore the difficulty of characterizing the impact of marijuana use in these patients while drawing attention to how risk may be present for some patients and not at all for others.

Several maternal characteristics have been consistently associated with increased risk, including maternal age, notably adolescents and women above the age of 30, and a maternal history of depression [1]. In particular, the risk of developing PPD was 20 times higher for women with a history of depression compared to those with no prior diagnosis of depression [7]. Among women experiencing PPD, studies also suggest a possible link between the experience of PPD and racial and ethnic minority status. From 2004 to 2012, the levels of PPD among minority groups remained more than double the rates in White, non-Hispanic new mothers [8]. These disparities between racial/ethnic groups and rates of PPD persist, even when controlling for socioeconomic factors [9]. Thus, pregnant women with any of these three risk factors may be predisposed to developing PPD.

In addition to racial and ethnic disparities, differences tied to socioeconomic status (SES) have also been identified as a major determinant of postpartum mental health risk disparities. Studies highlight that women of low SES tended to have increased depressive symptoms in late pregnancy and at two and three months postpartum. When quantifying SES risk indicators, women with all four risk indicators (low monthly income, less than a college education, unmarried status, and unemployed status) were 11 times more likely to have clinically elevated depression scores at three months postpartum compared to women with no SES risk indicators [10].

Despite consistent evidence that maternal age, minoritized racial/ethnic status, and prior mental health history predict PPD in general obstetric populations, it is not established whether these predictors retain prognostic value among individuals already at elevated risk by virtue of substance use in pregnancy. Clarifying this is clinically consequential, as it determines whether conventional risk stratification remains informative in this population or whether substance-exposed pregnancies warrant a distinct approach to postpartum surveillance.

The primary objective of this study was to determine whether maternal age, race/ethnicity, and pre-pregnancy mental health history independently predict PPD among individuals with biochemically confirmed tobacco, marijuana, and/or opioid use during pregnancy. The primary outcome was PPD, operationalized as at least one Edinburgh Postnatal Depression Scale (EPDS) [11] score ≥ 10 through six months postpartum and/or documented maternal report of a PPD diagnosis or treatment. It was hypothesized that older maternal age, minoritized racial/ethnic identity, and a documented pre-pregnancy mental health diagnosis would each be independently associated with increased odds of PPD after simultaneous adjustment for marital status, insurance type, adequacy of prenatal care utilization, and confirmed prenatal exposure to tobacco, marijuana, and opioids. It is important to note that several established psychosocial predictors of PPD, including intimate partner violence, social support, and perceived stress, were not available in the medical record and could not be modeled. The scope of this analysis is accordingly limited to predictors ascertainable from routine clinical documentation.

## Materials and methods

Data extraction**

This study involved a retrospective review of electronic medical charts from two academic pediatric practices in the Midwestern United States. The study included a racially, culturally, and geographically diverse sample of patients, with intentional oversampling of those who used tobacco, marijuana, and/or opioids during pregnancy. All substance use was determined by biochemical testing (maternal and infant urine drug screens, and cord blood analysis) and self-reported use. Participants with alcohol use or other non-target substances were excluded; alcohol exposure was not analyzed because prenatal alcohol use was not consistently ascertained via biochemical testing in the medical record, and reliance on self-report alone would likely introduce substantial exposure misclassification. Participants were limited to maternal-child dyads involving children born since July 2016 with pediatric medical records available through age three and linked maternal prenatal and delivery records.

All data collection for this study was performed via manual review of health system medical records for an extensive set of study variables for the parent study, including maternal medical and background factors and child outcomes. Abstraction was performed by trained abstractors using a standardized data dictionary and a structured electronic abstraction instrument, under the supervision of the project manager and principal investigator. Ambiguous cases were resolved by consensus with the principal investigator at recurring meetings. Patient dyads were included in the parent study if prenatal records were available for the woman, delivery records were available for both the woman and the child, and fewer than three well-check appointments were missing during the child's first year of life. For the current study, only women who used substances (tobacco, marijuana, and/or opioids) during pregnancy were included. Each substance (tobacco, marijuana, and opioids) was coded dichotomously as “any” or “no confirmed prenatal exposure.”

Biochemical confirmation of prenatal substance exposure was based on clinically indicated maternal urine drug screening, neonatal urine drug screening, and/or umbilical cord tissue analysis performed by the participating health systems' clinical laboratories. Self-report was applied only as an additional criterion for classification as exposed: individuals whose biochemical testing was negative but who disclosed use of a target substance during pregnancy were classified as exposed. Self-report was not used to exclude individuals with positive biochemical testing. Because biochemical testing was performed for clinical indications rather than on a standardized research schedule, testing was neither uniformly timed across pregnancy nor systematically repeated. Consequently, frequency of use, quantity consumed, duration of exposure, and trimester-specific exposure could not be reliably ascertained, and formal severity of substance use disorder was not consistently documented.

The parent Maternal-Child EMR project included 451 maternal-child dyads. Of those, 181 were excluded because they had no biochemically confirmed tobacco, marijuana, and/or opioid use during pregnancy. Analyses were conducted on complete cases. Of 270 eligible substance-exposed dyads, 15 (5.6%) were excluded for missing data on one or more primary variables (maternal age, race/ethnicity, pre-pregnancy mental health history, or the PPD outcome), yielding an analytic sample of 255.

The primary outcome was PPD in the mother. PPD was assessed using the EPDS, administered at pediatric well-child visits through six months postpartum at participating practices, and by maternal report of PPD diagnosis or treatment received outside our health system. A woman was classified as PPD-positive if she had ≥ 1 EPDS score ≥ 10 within this screening window and/or a maternal report of PPD diagnosis or treatment, including care received outside our health system, documented in the pediatric chart. Ascertainment of maternal PPD through the pediatric record reflects the recommendation of the American Academy of Pediatrics that maternal depression screening be integrated into the one-, two-, four-, and six-month well-child visits. Because attendance at well-child visits substantially exceeds attendance at postpartum obstetric visits, pediatric-record ascertainment was expected to capture a broader segment of the postpartum population than obstetric records alone would.

Primary predictors were maternal race/ethnicity, age, and mental health history, all of which were extracted from medical records. Additional demographic and medical information was extracted for the purposes of describing the study sample and exploring additional predictors of PPD. Prior mental health history was operationalized as any documented diagnosis of a mental health condition before the index pregnancy (e.g., depressive disorders, anxiety disorders, bipolar disorder, and trauma- and stressor-related disorders) as recorded in the maternal medical record.

Data analysis**

Descriptive analyses were used to describe the sample, and chi-square and t-test analyses were used to examine bivariate relationships between PPD and the other study variables. To determine which variables were most predictive of PPD, logistic regression analyses were conducted, with dichotomous PPD as the outcome and simultaneous entry of potential predictors, including maternal age, race/ethnicity, marital status, medical insurance status (income-dependent vs private; a marker of SES), prenatal care utilization, self-report and biochemically confirmed pregnancy substance use (tobacco, marijuana, and opioids), and a pre-pregnancy diagnosis of a mental health condition. Women with prior depression were not excluded, as prior mental health history is a clinically important predictor of PPD, and excluding these participants would reduce clinical representativeness and may bias estimates in this high-risk cohort.

Variables were coded as follows. Race/ethnicity was categorized as White non-Hispanic (reference), Black non-Hispanic, Hispanic, and Other or mixed race, with categories derived from documented self-identification in the medical record; the Other or mixed race category was composed predominantly of individuals identifying as Native American. Maternal age at conception was modeled in continuous years. Insurance was dichotomized as private, non-income-dependent versus income-dependent (Medicaid or equivalent), serving as a proxy indicator of socioeconomic position. Marital status was dichotomized as married or partnered versus unmarried. Each substance exposure and pre-pregnancy mental health history was coded as a dichotomous present/absent indicator. The outcome was dichotomous PPD, as defined above.

## Results

The study sample contained 451 cases that were part of the larger Maternal-Child EMR project. Of these, 270 cases used substances during pregnancy and were initially eligible for inclusion in this report. In the final analytic sample (N = 255), 77 women (30.2%) met the criteria for PPD. PPD diagnosis was evaluated using the child’s pediatric record and was defined as either a maternal report of a diagnosis of or treatment for PPD or at least one positive EPDS screen administered as part of a pediatric well-child visit by six months of age. A score of 10 or greater was considered positive. Table 1 shows the potential predictors of PPD.

**Table 1:** Differences in background characteristics by PPD status PPD: postpartum depression; SD: standard deviation

Variables	PPD absent (n = 178)	PPD present (n = 77)	t/χ2	P-value
Maternal age at conception, years, mean ± SD	28.5 ± 5.7	28.0 ± 5.9	0.610	0.540
Parity, mean ± SD	1.3 ± 1.3	1.3 ± 1.1	0.280	0.779
Pregnancy weight gain, pounds, mean ± SD	30.5 ± 19.4	32.6 ± 22.0	0.680	0.496
Health insurance, income-dependent, n (%)	150 (84.3%)	57 (74.0%)	3.00	0.083
Race/ethnicity, n (%)			4.16	0.245
White, non-Hispanic	92 (51.7%)	32 (41.6%)		
Black	56 (31.5%)	33 (42.9%)		
Hispanic	25 (14.0%)	8 (10.4%)		
Other/mixed	5 (2.81%)	4 (5.20%)		
Marital status, married/cohabitating, n (%)	99 (55.7%)	36 (46.8%)	1.73	0.188
Prenatal care, adequate, n (%)	96 (54.0%)	44 (57.1%)	0.150	0.703
Pregnancy marijuana use, n (%)	75 (42.1%)	47 (61.0%)	7.22	0.007
Pregnancy tobacco use, n (%)	91 (51.1%)	40 (51.9%)	0.020	0.904
Pregnancy opioid use, n (%)	57 (32.0%)	17 (22.1%)	2.58	0.108
Pre-existing mental health issue, n (%)	93 (52.2%)	45 (58.4%)	0.980	0.322

Participants who developed PPD were more likely to report marijuana use during pregnancy than those who did not develop PPD. Specifically, 61.0% of the PPD group reported marijuana use during pregnancy, compared with 42.1% of the no PPD group, and this difference was statistically significant (p < 0.007). All other background characteristics were similar between groups in unadjusted comparisons. Notably, race/ethnicity did not differ significantly between groups at the bivariate level but emerged as a significant predictor in the adjusted model. This pattern is consistent with confounding suppression, in which adjustment for covariates differentially distributed across racial/ethnic groups unmasks an association that was obscured in unadjusted analysis. Table 2 shows the logistic regression results for predictors of PPD.

**Table 2:** Logistic regression results predicting PPD from maternal background factors ^*^Indicates that the results are significant findings Full model R^2^ = 0.19, p = 0.011. Note: all predictors were simultaneously entered in the first step of the regression analysis, with the Adj. OR for each factor adjusted for all other predictors in the model PPD: postpartum depression; OR: odds ratio; CI: confidence interval

Predictor (comparator group)	Adj. OR	95% CI
Age	1.00	0.93–1.06
Race/ethnicity (ref = White, non-Hispanic)		
Black	2.57	1.21–5.47^*^
Hispanic	1.03	0.34–3.13
Other or mixed race	4.37	1.06–19.15^*^
Marital status (single, not cohabiting)	1.63	0.84–3.16
Medical insurance (private, non-income-dependent)	1.96	1.09–4.28^*^
Pregnancy weight gain	1.01	0.99–1.03
Prenatal care utilization (adequate)	1.13	0.57–2.18
Preexisting mental health condition (positive)	1.04	0.44–2.44
Pregnancy tobacco use (positive)	1.01	0.50–2.03
Pregnancy marijuana use (positive)	3.40	1.66–6.96^*^
Pregnancy opioid use (positive)	0.67	0.28–1.60

Overall, the model explained approximately 19% of the variance in PPD (R² = 0.19, p = 0.011). Three factors emerged as significant, independent predictors of PPD. Individuals with private medical insurance were nearly twice as likely to develop PPD as those with income-dependent insurance, and those who used marijuana during pregnancy were nearly three-and-a-half times more likely to develop PPD than those who did not. In addition, race was a significant predictor of PPD, with Black women being two and a half times more likely than White, non-Hispanic women to develop PPD, and women who reported another race or mixed race being more than four times more likely to develop PPD. After accounting for marijuana use, race/ethnicity, type of insurance, and the other factors in the model, having a prior mental health diagnosis did not increase the likelihood of developing PPD.

## Discussion

The current study provides important insights into the relationship between PPD and existing risk factors by examining how factors such as maternal race/ethnicity, age, and history of a mental health condition may contribute to the risk of developing PPD. 

Demographic factors

The study’s findings indicate that age did not significantly differ between women who did and did not experience PPD, suggesting that age may not increase PPD risk among individuals already at elevated risk due to substance use during pregnancy. However, race and ethnicity emerged as a significant independent predictor of PPD. Compared to White, non-Hispanic women, Black women had more than 2.5 times higher odds of experiencing PPD, while women identifying as Other or Mixed race had nearly 4.5 times higher odds of experiencing PPD. Hispanic women, however, were not associated with increased odds. Notably, the “Other or Mixed race” category was composed predominantly of women identifying as Native American, a group that is substantially underrepresented in perinatal mental health research. This estimate rests on a small subgroup with a wide confidence interval, however, and should be regarded as hypothesis-generating rather than as a stable effect estimate. 

These results are consistent with a larger cross-sectional study of 122,995 postpartum individuals that reported significant racial and ethnic disparities in PPD, with Black individuals at the highest risk [12]. Similarly, in a study examining racial and ethnic differences in PPD symptoms across the US, people identifying as Asian, Native Hawaiian, Pacific Islander, Southwest Asian, Middle Eastern, North African, those with mixed racial and ethnic identities, or those identifying as Black experienced the highest prevalence of early PPD symptoms [13]. This study highlights that women of different ethnic backgrounds are already at a disproportionately elevated risk of developing PPD based on their race and/or ethnicity. These studies further emphasize the need to consider structural and institutional contributors to PPD that may augment the risk that already vulnerable groups face when exposed to substances.

Socioeconomic factors**

When examining socioeconomic factors, the current study identified a significant association between health insurance type and PPD among individuals who used substances during pregnancy. Women with private, non-income-dependent insurance were nearly twice as likely to develop PPD compared to those with income-dependent insurance in the sample. This unexpected result diverges from a retrospective cohort study of more than 1.5 million participants, in which PPD prevalence was 7% among privately insured women compared to 13% among those with Medicaid [14]. One possible explanation is that private insurance serves as a proxy for higher SES, which may be associated with unique stressors that increase PPD risk. Women with higher SES may face greater pressure to balance professional and social roles, have fewer external support systems, or encounter stigma when seeking mental health care despite greater financial resources.

In addition, these women may have to return to work sooner, which can add further stress to the postpartum period and increase time away from their newborn, thereby increasing the risk for PPD [15]. Consistent with this, Kornfeind and Sipsma also reported that many mothers are unable to stay home as long as they would like, most commonly due to financial constraints or exhaustion of leave benefits, and among women taking 12 weeks of leave or less, each additional week of maternity leave was associated with lower odds of PPD symptoms [15]. This unexpected association contrasts with prior evidence and may reflect differences in ascertainment rather than true differences in risk. 

Privately insured individuals in this cohort may have had greater access to depression screening and mental health services outside the health system, generating more opportunities for a PPD diagnosis to be documented. Conversely, publicly insured individuals may have experienced true PPD that went unscreened, undiagnosed, or unrecorded. Interpreting this finding as evidence that higher socioeconomic position confers PPD risk would be unwarranted. We report it because differential detection is itself a clinically actionable problem, but we caution explicitly against its use to guide risk stratification. Consistent and equitable screening remains essential for all postpartum patients, regardless of insurance status. 

Additionally, while private insurance often indicates better access to healthcare, it does not guarantee comprehensive mental health coverage, and some plans impose higher out-of-pocket costs compared to Medicaid, which could discourage treatment. Indeed, research has shown that postpartum healthcare costs are significantly higher for individuals with commercial insurance compared to those with Medicaid. One study conducted in Colorado found that total adjusted per-person spending was $1,110 higher for those with private insurance, with out-of-pocket costs averaging $796 more than for Medicaid patients [16]. These increased financial burdens may discourage postpartum individuals from seeking necessary mental health care.

In contrast, Medicaid’s lower cost-sharing requirements and broader coverage of postpartum services may facilitate better access to mental health care, potentially leading to earlier identification and management of PPD in Medicaid-insured populations. Although specific comparative information for these types of coverage in Michigan is not readily available, this overall trend may partially explain why women with private insurance in our study exhibited a higher risk for PPD despite their presumed greater access to healthcare services [16]. The higher rates of PPD in women with private insurance in the sample may also be explained by PPD rates being less frequently identified in women with insurance that was not private. Research has shown that primary care providers order fewer preventive services for women with Medicaid compared to those with private insurance [17].

These Medicaid-insured individuals were more likely to be younger, non-White, and living in areas with higher poverty rates, all of which are factors that could influence healthcare utilization. These disparities were particularly evident in OB/GYN visits, highlighting the need to prioritize maternal healthcare. Although some studies have found no significant difference in depression screening rates between Medicaid and non-Medicaid patients at postpartum visits, additional research is needed to understand how the frequency of preventive services offered may still vary by insurance type [18]. One proposed solution involves implementing a new payment model that incentivizes physicians to prioritize preventive care for PPD. Under this program, physicians were found to be twice as likely to conduct PPD screenings [19]. Thus, although women with lower incomes may experience higher rates of PPD, the absence of private insurance could mean their PPD is less frequently identified, and implementing payment models that incentivize physicians to prioritize preventive care may help address this finding.

Health and lifestyle factors

This analysis also evaluated multiple health and lifestyle factors during pregnancy. Neither parity nor pregnancy weight gain differed significantly between those who developed PPD and those who did not. Pre-pregnancy mental health history was associated with an increase in the odds of PPD, but this did not reach statistical significance in the adjusted model. This should be interpreted as a non-significant finding in a modestly sized, highly selected sample rather than as evidence of no association. Three features of our measurement argue for conservatism. Psychiatric history was captured as a single binary indicator that collapsed conditions of markedly different type, severity, chronicity, and treatment status; documented psychiatric diagnosis in the medical record substantially under-ascertains true psychiatric history, particularly among individuals with fragmented care; and psychiatric history is strongly correlated with substance use in this population, such that simultaneous entry of both may distribute shared variance in ways that obscure an independent effect. The point estimate remained elevated, with a confidence interval encompassing clinically meaningful effects.

Given the strong and consistent prior evidence that depression history predicts PPD [2,7], our null result is best understood as a limitation of this analysis rather than as a challenge to that literature, and it should not be used to justify deprioritizing psychiatric history in postpartum risk assessment. Future studies should further investigate these relationships to inform targeted interventions aimed at reducing PPD risk in high-risk populations.

Substance use

Among pregnant individuals who used substances, marijuana use during pregnancy was the strongest independent predictor of PPD in this sample. The mechanism underlying this association cannot be determined from these data, and at least two explanations are compatible with our findings. Cannabis has psychoactive properties that could plausibly influence maternal mood through effects on endocannabinoid signaling and autonomic regulation. Equally plausible, however, is reverse causation or confounding by indication: individuals experiencing subthreshold depressive or anxious symptoms during pregnancy may use cannabis to self-medicate, in which case prenatal cannabis use may function as a marker of underlying affective vulnerability rather than as a cause of subsequent depression.

This interpretation is consistent with the findings of previous research, which observed no clear temporal association between prenatal cannabis use and trajectories of depressive symptoms [20]. Because prenatal depressive symptoms were not systematically assessed in our cohort and the temporal sequence of symptom onset relative to cannabis use could not be established, we cannot adjudicate between these explanations. Our finding should therefore be read as identifying prenatal cannabis use as a clinically useful risk marker warranting intensified postpartum surveillance, not as evidence that cannabis exposure causes PPD.

By contrast, tobacco and opioid use during pregnancy did not significantly predict PPD in this study. This may be due to factors such as substance use behaviors and their varied psychological and physiological impacts on mental health among pregnant women. However, these associations should be interpreted in the context of cohort selection and documentation practices. Because the parent chart-review cohort intentionally oversampled substance-exposed pregnancies and required linked maternal-child records with pediatric follow-up, the analytic sample may not represent all pregnant individuals who use substances. Additionally, differential screening, disclosure, access to care, or documentation by socioeconomic strata could influence which cases of PPD are identified in the medical record, potentially contributing to the observed association between private insurance and PPD.

Strengths and limitations

This study has several strengths. Prenatal substance exposure was ascertained biochemically rather than by self-report alone, avoiding the substantial under-disclosure that characterizes self-reported substance use in pregnancy, particularly where disclosure carries perceived legal or child-protective consequences. The outcome was assessed using a validated instrument administered within a clinical workflow with high population reach. The cohort was racially and socioeconomically diverse and deliberately enriched for substance exposure, permitting examination of predictors within a high-risk population typically too sparsely represented in general obstetric samples to analyze separately. Finally, linkage of maternal prenatal and delivery records to longitudinal pediatric records established clear temporal ordering between prenatal exposure and postpartum outcome.

However, the study has certain limitations as well. Formal inter-rater reliability statistics were not calculated for chart abstraction. Although abstractors worked from a standardized data dictionary with supervised training and consensus adjudication of ambiguous cases, the absence of a quantified agreement statistic means that abstraction error cannot be excluded as a source of measurement variability, particularly for variables requiring judgment, such as pre-pregnancy mental health history.

Prenatal alcohol exposure was not systematically ascertained with biochemical testing in the medical record and was therefore excluded from the analytic sample. Accordingly, these findings may not be generalizable to pregnancies primarily exposed to alcohol. In addition, results should be interpreted in the context of cohort selection. The analytic sample was derived from a parent chart-review cohort designed to oversample substance exposure and was further restricted by eligibility requirements and exclusions, including non-target substances and incomplete primary-variable data. These design features may limit generalizability to broader pregnant populations and settings with different screening and ascertainment practices.

EPDS screening in our participating practices was administered during pediatric well-child visits through six months postpartum and was not sufficiently consistent beyond this period to evaluate symptom trajectories or persistence across the full first year. As a result, our outcome definition, which included maternal report of diagnosis or treatment for PPD and/or at least one EPDS score ≥ 10, captures probable PPD or clinically elevated depressive symptoms in the early postpartum period, but it may include transient symptoms and does not distinguish persistent from episodic depression or depression beyond six months postpartum. This limitation may attenuate associations if persistent depression has different risk factors than a single elevated screen. Additionally, although we captured the presence of a pre-pregnancy mental health diagnosis, we could not consistently distinguish specific conditions, symptom severity, or treatment history across records, which may contribute to residual confounding.

Prenatal substance exposure was necessarily characterized as a dichotomous any-or-none indicator for each substance. We could not quantify frequency, quantity, duration, or trimester of exposure, nor grade the severity of substance use disorder. Dichotomous classification groups light/first-trimester-only exposure with heavy, sustained use; because such non-differential misclassification generally biases estimates toward the null, the true magnitude of the marijuana-PPD association may be underestimated. Polysubstance exposure was addressed through mutual adjustment rather than by modeling exposure patterns, and the sample was insufficiently powered to characterize interactions among substances.

Several of the most consistently replicated predictors of PPD could not be evaluated. Intimate partner violence, perceived social support, perceived stress, educational attainment, employment status, and housing or food insecurity were not systematically documented in the available records. These factors are not randomly distributed: each is patterned by both racial/ethnic identity and socioeconomic position through structural mechanisms, including differential exposure to discrimination, neighborhood disadvantage, and caregiving burden. Residual confounding by these unmeasured factors is therefore likely and may account for some or all of the associations we observed with race/ethnicity and insurance type. Our findings should be understood as identifying subgroups with elevated observed PPD risk in this cohort, not as identifying the causal mechanisms producing that risk.

Estimates for smaller racial and ethnic subgroups were imprecise. The Other or Mixed race category included nine participants (aOR: 4.37, 95% CI: 1.06-19.15), and the Hispanic category included 33 participants (aOR: 1.03, 95% CI: 0.34-3.13). Because of these small subgroup sizes and wide confidence intervals, the Other or Mixed race finding should be considered hypothesis-generating, and the lack of a significant association among Hispanic participants should not be interpreted as evidence of equal risk.

Two forms of bias merit specific attention. First, because the parent cohort deliberately oversampled substance-exposed pregnancies and required linked maternal-child records with pediatric follow-up through age three, individuals who disengaged from care, relocated, or experienced custody disruption are systematically underrepresented; these are plausibly individuals at elevated rather than reduced risk of PPD, and their absence may bias prevalence downward. Second, biochemical testing was clinically indicated rather than universal, and there is well-documented variation in who is tested during pregnancy and delivery, with testing applied disproportionately to publicly insured and Black patients. Differential testing means the substance-exposed cohort may itself be non-representative of all substance-exposed pregnancies, and this ascertainment pattern is a plausible contributor to the counterintuitive insurance association we observed.

Finally, we also recognize that PPD was not a rare outcome in this cohort. When outcomes are common, odds ratios can overstate the magnitude of effect compared with risk ratios, and thus the strength of associations may appear larger than the corresponding relative risks.

## Conclusions

This study underscores the multifaceted nature of PPD among pregnant individuals who use substances, revealing that marijuana use, race/ethnicity, and private insurance status are significant independent predictors of PPD risk. In contrast, while age and preexisting mental health conditions were associated with higher PPD rates, these factors did not reach statistical significance in the final model, reflecting complex interrelationships among income, mental health history, and substance use. The higher observed PPD incidence among women with private insurance may reflect barriers to recognition, diagnosis, or treatment among lower-income women, but this finding should be interpreted cautiously. Because the analytic sample was derived from a retrospective cohort designed to oversample substance-exposed pregnancies and required linked maternal-child records with pediatric follow-up, the association may also reflect selection or ascertainment effects, including differential screening, disclosure, access to care, or documentation across socioeconomic groups.

Moreover, social determinants of health were incompletely captured in our records. Beyond insurance type, marital status, and adequacy of prenatal care utilization, more granular factors (e.g., education, employment, housing/food insecurity, neighborhood context, and history of interpersonal violence) were not consistently available, which may have contributed to residual confounding. These findings emphasize the importance of ongoing and comprehensive screening for PPD in prenatal and postnatal care, particularly for women who use marijuana or who have socioeconomic advantages that might obscure mental health vulnerabilities. Future large-scale, diverse, and longitudinal research is needed to further clarify how socioeconomic and clinical factors intersect with substance use to shape PPD risk. Addressing these key predictors through targeted prevention and early intervention strategies offers a pathway toward improving maternal and child outcomes in high-risk populations.
