# Correction: Postpartum Depression Following a Substance-Exposed Pregnancy: The Role of Age, Race/Ethnicity, and Mental Health History

**DOI:** 10.7759/cureus.c500

**Published:** 2026-11-05

**Authors:** Natalie Aguilar, Miranda C Manzo, Patrick Fakhoury, Jacob Surma, Beth Bailey

**Affiliations:** 1 Pediatrics, Central Michigan University College of Medicine, Saginaw, USA; 2 Obstetrics and Gynecology, Central Michigan University College of Medicine, Saginaw, USA; 3 Anesthesiology, Central Michigan University College of Medicine, Mount Pleasant, USA; 4 Otolaryngology, Central Michigan University College of Medicine, Mount Pleasant, USA; 5 College of Medicine, Central Michigan University College of Medicine, Mt. Pleasant, USA

This article has been corrected to add a fifth and final author as well as a funding statement that was omitted from the original published version.

